# Daytime Sleepiness, Apnea, Neuroimaging Correlates and Cortisol Dysregulation in a Memory Clinic Cohort

**DOI:** 10.14283/jpad.2024.145

**Published:** 2024-07-18

**Authors:** Charlotte Sørensen, I. Kåreholt, G. Kalpouzos, C. T. Udeh-Momoh, J. Holleman, M. Aspö, G. Hagman, G. Spulber, M. Kivipelto, A. Solomon, S. Sindi

**Affiliations:** 1 Division of Clinical Geriatrics, Department of Neurobiology, Care Sciences, and Society, Karolinska Institutet, Stockholm, Sweden; 2 Aging Research Center, Karolinska Institutet and Stockholm University, Stockholm, Sweden; 3 Institute of Gerontology, School of Health and Welfare, Jönköping University, Jönköping, Sweden; 4 Ageing Epidemiology Research Unit (AGE), School of Public Health, Faculty of Medicine, Imperial College London, London, UK; 5 Global Brain Health Institute, University of California San Francisco, San Francisco, USA; 6 Brain and Mind Institute, Aga Khan University, Nairobi, Kenya; 7 Theme Inflammation and Aging, Karolinska University Hospital, Stockholm, Sweden; 8 Institute of Public Health and Clinical Nutrition, University of Eastern Finland, Kuopio, Finland; 9 Institute of Clinical Medicine, Neurology, University of Eastern Finland, Kuopio, Finland; 10 Karolinska Vägen 37A, 17164 Solna, Sweden

**Keywords:** Self-reported sleep, neuroimaging, Cortisol, cognitive impairment

## Abstract

**Background:**

Sleep disturbances as well as cortisol hypersecretion are increasingly acknowledged as risk factors for Alzheimer’s disease (AD). However, the mechanisms underlying the association, and the interplay with cortisol abnormalities, remain unclear.

**Objectives:**

This study aims to identify how self-reported sleep disturbances are associated with structural brain measures and diurnal cortisol dysregulation among memory clinic patients.

**Design:**

A cross-sectional study performed at Karolinska University Hospital Memory Clinic, Sweden.

**Participants:**

The study was based on 146 memory clinic patients diagnosed with either subjective cognitive impairment or mild cognitive impairment.

**Measurements:**

Self-reported sleep was measured using the Karolinska Sleep Questionnaire. MRI or CT was used to quantify structural brain measures using four visual rating scales (Scheltens, Pasquier, Koedam, and Fazekas scales), and salivary cortisol was sampled to measure diurnal cortisol patterns through measures of cortisol immediately after awakening, cortisol awakening response, bedtime cortisol, total cortisol from awakening to bedtime, and the AM/PM cortisol ratio.

**Results:**

Increased sleep apnea index (OR=1.20, 95% CI=1.04:1.39, p=0.015) was associated with greater odds of posterior brain atrophy, measured by the Koedam visual rating scale, and reduced awakening Cortisol (β=−0.03, 95% CI=− 0.07:0.00, p=0.045). Increased daytime sleepiness was associated with both reduced awakening cortisol (β=−0.03, 95% CI=−0.06:0.00, p=0.025) and a reduced AM/PM cortisol ratio (β=−0.04, CI=−0.08:−0.01, p= 0.021).

**Conclusion:**

In a memory clinic cohort self-reported sleep disturbances are associated with both worse structural brain tissue integrity and altered diurnal cortisol profiles. These findings may add insights into possible mechanisms behind sleep disturbances in aging with subjective and cognitive impairment.

**Electronic Supplementary Material:**

Supplementary material is available for this article at 10.14283/jpad.2024.145 and is accessible for authorized users.

## Introduction

**S**leep disturbances are increasingly acknowledged as a risk factor for Alzheimer’s disease (AD) (1). A meta-analysis of 18 longitudinal studies found that overall sleep problems, insomnia, sleep-disordered breathing (SDB), sleep-related movement disorder, sleep pattern change, and nonspecific sleep problems, all are associated with an increased risk of developing AD in older adults (2). However, sleep was not included in the latest report on dementia prevention stating 12 potentially modifiable risk factors that may account for 40% of all dementia incidents (1). Thus, while sleep disturbances are increasingly acknowledged as a risk factor for AD, the underlying mechanisms remain unclear (1, 3–6).

Sleep is important for brain homeostasis and neuroplasticity (7). This has led several cross-sectional studies to investigate the association between sleep disturbances and brain structure. It is well established that sleep is important for memory (8), and the hippocampus is therefore a highly investigated brain structure in sleep research. An early study by Riemann and colleagues found that insomnia patients had lower hippocampal volume compared to participants without insomnia (9). While some studies have also found lower hippocampal volume in insomnia patients (10) and in people with excessive daytime sleepiness and fatigue (11), other studies failed to reproduce this association in insomnia patients (12–16). Furthermore, insomnia patients have lower grey matter volume (GMV) in various areas of the frontal lobe (12, 17–19), parietal lobe (12, 17) and the cingulate cortex (17), while GMV is larger in the cingulate cortex (16, 20).

Obstructive sleep apnea (OSA) has also been heavily researched for its associations with brain measures (21, 22). Both insomnia patients (17, 19) and patients with OSA (22, 23) show lower GM in the temporal lobe. For OSA patients, the oxygen desaturation upon OSA is associated with reduced cortical thickness in the temporal lobe, whereas the sleep disturbance component of OSA is associated with increased cortical thickness in the parietal and frontal lobe (22). Further, It has been found that OSA is associated with lower white matter diffusivity (i.e. white matter integrity) and that this is already found upon mild OSA compared to healthy controls (21). Lastly, poor sleepers without a sleep diagnosis have reduced GMV in the parietal lobe (24).

Overall, much uncertainty still exists about the relation between sleep and brain morphological changes, and while many studies have been conducted among younger and middle-aged patients with sleep disorders, especially insomnia and OSA, less is known about how general sleep disturbances associate with brain morphology in older memory clinic participants.

Sleep disturbances have been reported to be associated with cortisol dysregulation (25–27). The stress hormone cortisol is the end product of the Hypothalamic-Pituitary-Adrenal (HPA) axis, and the interaction between the HPA-axis and sleep are both highly complex and bidirectional (26, 28). Hyperactivity of the HPA axis has been linked to decreased sleep duration/quality (27, 28). However, findings on the associations between sleep and HPA-axis diverge highly due to differences in sampling method of cortisol (urine, blood, or saliva), the time of sampling (27), and severity of sleep disturbances. A meta-analysis found insomnia patients to have moderate higher cortisol levels both during the night and day compared to controls of good sleepers (27). Insomnia patients had higher levels of cortisol before bedtime (samples between 20:00 – 23:00) and during the night (samples between 23:30 – 06:30) compared to good sleepers (27). This link between sleep and HPA hyperactivity is further shown in observational studies where insomnia patients have increased 24h cortisol levels (29), increased evening and nocturnal cortisol levels (30), and increased awakening cortisol upon short sleep duration (total sleep time <5h) (31). In older adults (62–90 years) greater sleep fragmentation and increased wakening-after-sleep-onset have been associated with higher daytime cortisol levels (32). Furthermore, high cortisol levels are, besides being associated with sleep, also associated with worse memory and smaller hippocampal volume (33). A study by Basta and colleagues found that older adults living with mild cognitive impairment (MCI) have higher morning cortisol levels compared to cognitively healthy older adults (34). Additionally, Basta and colleagues found that participants with MCI, who also experience insomnia, have even higher morning cortisol levels than those without insomnia (34). However, some studies have shown no difference in cortisol levels between patients with and without insomnia (35, 36), while others have shown decreased awakening cortisol in insomnia patients (37). In summary, sleep and the HPA-axis have a bidirectional relationship that is still not fully understood.

Evidence on the interplay between sleep disturbances, cortisol dysregulation and AD development have been inconclusive across studies. There is a large variety of sleep disturbances and assessment procedures, cortisol sampling methods and timing, which together with different cohort characteristics, lead to divergent results. Also, previous studies have primarily involved diagnosed insomnia patients, even though a meta-analysis showed that various types of sleep disturbances are associated with a higher risk of dementia, including AD (2).

The aim of this study is to investigate the association between self-reported sleep disturbances, diurnal cortisol profiles, and their interplay, and how they associate with neuroimaging correlates, in a memory clinic cohort of middle aged to older adults. Based on previous studies we expect that poor sleep is associated with lower brain tissue integrity (measured through scales of atrophy and white matter hyperintensities) and dysregulation of diurnal cortisol (using five measures reflecting diurnal cortisol profiles).

## Methods

### Subjects

The study sample is based on the cohort study Cortisol and Stress in Alzheimer’s Disease (Co-STAR) at the Karolinska University Hospital memory clinic in Huddinge, Sweden, from 2014 to 2017. The cohort was previously described by Holleman and colleagues (38). To be included in the study, participants had to be above 45 years of age and not have any condition affecting the HPA-axis. Of the invited participants, a total of 233 subjects fulfilled the inclusion criteria and wished to participate in the study, and 188 participants provided sufficient data to be included in the cohort. At baseline, participants were diagnosed with subjective cognitive impairment (SCI), MCI, or AD. Due to the use of self-reported sleep measures, which rely on memory capacities, only participants diagnosed with SCI and MCI were included in the current analysis. This provided a final sample size of 146 participants.

### Procedure

All participants underwent a clinical assessment that included, among others, a neuropsychological test battery at baseline, sleep questionnaire, MRI or CT brain imaging, and saliva cortisol measures, as part of a baseline assessment.

### Clinical assessment

For the clinical assessment, all participants met with neuropsychologists at the Karolinska University Hospital memory clinic in Huddinge for a comprehensive cognitive assessment. The cognitive tests performed were similar to standard clinical practice and covered the cognitive domains; memory, working memory, processing speed, perceptual reasoning and overall cognition. For more information see the article by Holleman and colleagues (38) where each cognitive test is presented in more detail. Participants were diagnosed with MCI, SCI or AD based on commonly used diagnostic criteria. AD was diagnosed based on the International Classification of Diseases-10 (ICD-10) dementia criteria (39), and MCI was diagnosed according to the criteria of Winblad and colleagues (40). Lastly, participants who had been referred to the memory clinic by the general practitioner for reporting subjective cognitive impairments, but did not reach the criteria of Winblad and colleagues nor the ICD-10 dementia criteria, were diagnosed with SCI. The criteria for SCI is consistent with common practice, which includes two major features (41). First, a self-experienced decline in cognitive capacity, compared with a previously normal cognitive status, and second, normal performance on standardized cognitive tests used to classify MCI, adjusted for age, sex, and education (41).

### Sleep questionnaire

Self-reported sleep measures were obtained using the Karolinska Sleep Questionnaire (KSQ) (42). The KSQ is a commonly used and validated questionnaire for self-reported sleep (42). The questionnaire is composed of 18 items (items a – r) that reflect both nocturnal sleep disturbances and daytime sleepiness (42). For each item, the question asked is “have you had this sleep problem during the last three months”. Participants then rate the questions with a score between 0 and 5, where 0 = Never, 1 = Seldom (few times/year), 2 = Sometimes (several times/month), 3 = Often (1–2 times/week), 4 = mostly (3–4 times/week) or 5 = Always (+5 times/week). Thus, a high score depicts worse sleep. Several sleep disturbances were identified using the full KSQa-r (Table 1). For some sleep parameters we were able to use individual KSQ items, while for others the parameter consists of several KSQ items. Secondly, in line with previous research (42) on the KSQ, we have used the four sleep indexes; daytime sleepiness (only in leisure time), sleep quality, non-restorative sleep, and sleep apnea index. All sleep disturbance parameters and sleep indexes are displayed in Table 1 with their KSQa-r items and their ordinal scale.

**Table 1:** Sleep disturbance parameters and sleep index measures used in the study

**Sleep disturbances**	**KSQ item**	**Range Theoretically**	**Range In cohort**
Total insomnia	a + c + i	[0–15]	[0–15]
Sleep quality	h + j + m	[0–15]	[0–15]
Sleep apnea	e + f	[0–10]	[0–10]
Daytime sleepiness (during work time)	n + p	[0–10]	[0–10]
Global sleep	All 18	[0–90]	[0–73]
Initial insomnia	a	[0–5]	[0–5]
Middle insomnia	c	[0–5]	[0–5]
Terminal insomnia	i	[0–5]	[0–5]
Insufficient sleep	l	[0–5]	[0–5]
Difficulties waking up	b	[0–5]	[0–5]
Nightmares	g	[0–5]	[0–5]
Restless leg	k	[0–5]	[0–5]
Snoring	d	[0–5]	[0–5]
General sleep	u *	[0–4]	[0–4]
Sleep indexes			
Daytime sleepiness	o + q + r	[0–15]	[0–15]
Sleep quality index	a + c + I + j	[0–20]	[0–20]
Non-restorative sleep index	b + h + m	[0–15]	[0–15]
Sleep apnea index	d + e + f	[0–15]	[0–15]

All sleep variables are based on the Karolinska Sleep Questionnaire (KSQ). u* = Question: “how is your sleep in general?”. Higher scores indicate more sleep disturbances.

### Neuroimaging scales

Four neuroimaging visual rating scales were used to determine structural brain measures, based on brain scans, acquired by either CT or MRI, of each participant. Each imaging scale is sensitive to different brain regions where changes are known to occur during the course of dementia (43). All the scales are validated and commonly used both in research and in clinical settings (43, 44). The four scales used are: Medial Temporal Atrophy (MTA), Cortical Global Atrophy (GCA), White Matter Hyperintensities (WMH) and Posterior Atrophy (PA). The MTA scale, also referred to as the Scheltens scale, is a 5-point (0–4) scale measuring the atrophy in the temporal lobe by assessing the height of the hippocampus and the width of the temporal horns and the choroid fissure (44, 45). The GCA scale by Pasquier assesses global cerebral atrophy (46). It is a 4-point (0–3) scale that visually measures the loss of gyral volume and widening of the sulci in 13 different brain regions (44). The PA scale, also known as the Koedam scale, measures the extent of posterior atrophy in the posterior cingulate sulcus, precuneus, parieto-occipital sulcus and the parietal cortex with a 4-point (0–3) rating scale (47). Lastly, WMH, the Fazekas scale, rates deep white matter hyperintensities and periventricular hyperintensities using a 4-point (0–3) scale (48). All four scales can be assessed on both CT and MRI (44, 49), and for both WMH, MTA, and GCA the intramodality agreement between CT and MRI is high (mean weighted k from three observers; GCA=0.83, MTA = 0.88, WMH = 0.79) (49). Further, no systematic trend to rating one modality higher than the other was found (49). Lastly, all are imaging scales are ordinal, where higher scores indicate greater atrophy (or lower tissue integrity in cross-sectional studies) (44).

### Cortisol measures

Each participant received a home kit to self-measure salivary cortisol. Participants were instructed to sample saliva on 2 non-consecutive weekdays, at 6 exact times points during the day: Immediately after awakening in the morning (T1), 30 minutes post-awakening (T2), 1-hour post awakening (T3), at 14.00 o’clock (T4), at 16.00 o’clock (T5), and right before bedtime (T6). Participants documented the exact sampling time point and kept the samples in their freezer until sending them to the clinic for analysis. Subsequently, the clinic sent all samples to Dresden LabService GmbH for analyses. Further details are described in previous work (38).

For analyses, all six daily cortisol samples were first checked to see if they were collected at the instructed time points. The diurnal pattern of cortisol varies greatly in the morning post awakening, giving great importance to the sampling time (50). Therefore, we constricted the morning samples at T1, T2, and T3 to have a maximum offset of ±15 minutes from the instructed time of measurement. This restriction led to dismissal of the T3 measure, due to a large proportion of participants who had invalid data for the T3 measurement on both sampling days. Secondly, all cortisol measures on both sampling days were combined by averaging the measures of the two sampling days. The combined scores were then winsorized such that values were limited to 3 SD from the mean, to reduce the influence of outliers (50). Based on the five daily cortisol measures, we computed the following measures to estimate the diurnal cortisol pattern: awakening cortisol, cortisol awakening response (CAR), bedtime cortisol, total daily cortisol, and the AM/PM cortisol ratio. For awakening and bedtime cortisol levels, we then used the combined T1 and T6 measures, respectively, winsorized at ±3SD. Therefore, awakening cortisol reflects the cortisol levels immediately after awakening. The CAR measure reflects the increase in cortisol levels from awakening to 30 minutes post awakening, measured by the area under the curve with respect to increase (AUCi) from T1 to T2 (51). The total daily cortisol output was calculated as the area under the curve with respect to ground (AUCg) for all five timepoints (51). Lastly, the AM/PM cortisol ratio was calculated by dividing the winsorized T1 by T6. To account for non-normality both the awakening and bedtime cortisol measures were log transformed, and the daily cortisol and AM/PM cortisol ratio have been zero-skewness ln transformed.

### Statistical analysis

Descriptive statistics are reported as proportions (N,%) for categorical data and means±SD for continuous and ordinal data. Assessment of the data distribution was performed both graphically and by using the Shapiro-Wilk test of normality. For groupwise comparisons of continuous data following a normal distribution, student t-tests were performed to detect mean differences between the SCI and MCI groups. For ordinal data and continuous data not following a normal distribution, non-parametric Wilcoxon rank sum tests were performed. For categorical data (sex, smoking and sleep medication) chi-squared tests (*Χ*^2^) or Fisher’s exact test (alcohol consumption) was performed. All tests were two-sided and with a significance level of 0.05.

Ordinal logistic regressions were performed to explore the relationship between sleep measures and structural brain measures. Each imaging scale (MTA, GCA, WMH, and PA) was treated as an ordinal outcome variable in the regression models, and each KSQ item was treated as a numeric predictor. All regression models were controlled for age, sex, education, and alcohol consumption, as these factors are associated with sleep and dementia risk (5, 52). Results are presented as odds ratios (OR), 95 % confidence intervals (CI), and p-values. Secondly, all significant associations between sleep and visual rating scales were modeled with cortisol (Morning, Bedtime, Total cortisol, CAR and AM/PM ratio) as an interaction term.

A series of linear regression models were carried out to explore the relationship between sleep measures and the diurnal cortisol pattern. All models were controlled for age, sex, education and alcohol consumption, and results were presented as β-coefficients, 95 % CI, and p-value. Both the ordinal logistic regressions and linear regressions were adjusted for age, sex, education, and alcohol consumption.

Previous studies show that females and males have different sleep disturbances (53), therefore all significant associations were additionally examined for sex differences by stratifying for sex. For the ordinal logistic regression we only controlled for age and education due to the reduced sample size upon stratification.

All significant regression models (non-stratified) were further adjusted for depressive symptoms evaluated by GDS-15 (Geriatric Depression Scale) and if the participant took any sleep medication.

All statistical analyses were performed in R (4.2.1) statistical software, using the MASS package.

## Results

### Participants

Characteristics of the Co-STAR cohort are shown in Table 2. The SCI and MCI groups did not differ significantly in demographic factors, structural brain measures (measured by four visual rating scales), diurnal cortisol (measured by five computed cortisol measurements), or in sleep parameters (measured by the KSQ) (all p≥0.05, Table 2). The total sample analyzed comprises 146 participants of whom 58 % were female (N=84). The mean age of the whole sample was 61±7 years (mean ± SD), ranging from 47 to 86 years of age.

**Table 2:** Sample characteristics of selected variables in the Co-STAR study

	**N**	**Whole sample (N=146)**	**SCI (N=68)**	**MCI (N=78)**	**p-value**
**Demographics**					
Sex (Female ratio; N,%)^a^	146	84 (58%)	41 (60%)	43 (55%)	0.529
Age (years, mean (SD))^b^	146	61 (7)	60 (6)	62 (8)	0.261
Range		47 – 86	47 – 75	47 – 86	
Education (years, mean (SD))^c^	146	14.1 (3.3)	14.6 (3.3)	13.8 (3.3)	0.151
Range		7 – 26	8 – 26	7 – 22	
Alcohol consumption (N,%)^d^	144				0.194
Abstaining		12 (8.3%)	4 (5.9%)	8 (11%)	
Normal consumption		127 (88%)	60 (88%)	67 (88%	
(history of) Alcohol abuse		5 (3.5%)	4 (5.9%)	1 (1.3%)	
Smoking (N, %)^a^	140				0.765
Never smoked		50 (36%)	24 (37%)	26 (35%)	
Previous smoker		73 (52%)	32 (49%)	41 (55%)	
Current smoker		17 (12%)	9 (14%)	8 (11%)	
Depressive symptoms (mean, SD)^b^	126	5.9 (4.0)	5.1 (3.5)	6.5 (4.4)	0.105
Sleep medication (N,%)^a^	145				0.313
Do not take medication		121 (83%)	59 (87%)	62 (81%)	
Take medication		24 (17%)	9 (13%)	15 (19%)	
**AD Biomarkers (mean, SD)**^b^ **[ng/L]**	124				
Amyloid beta		801 (216)	862 (184)	746 (229)	0.002
Total tau		292 (147)	272 (112)	310 (172)	0.389
Phosphor tau		42 (18)	40 (14)	44 (20)	0.575
**Neuroimaging correlates (mean, SD)**^c^					
MTA [0–4]	143	0.76 (0.64)	0.63 (0.48)	0.86 (0.74)	0.134
GCA [0–3]	140	0.61 (0.50)	0.52 (0.44)	0.69 (0.54)	0.098
PA [0–3]	73	0.34 (0.49)	0.31 (0.43)	0.38 (0.55)	0.865
WMH [0–3]	133	0.73 (0.72)	0.71 (0.59)	0.75 (0.82)	0.603
**Cortisol (mean, SD)**^c^					
Awakening cortisol (nmol/l)	124	8.66 (4.88)	8.25 (4.47)	9.07 (5.24)	0.376
Bedtime cortisol (nmol/l)	124	2.34 (3.68)	1.96 (2.70)	2.71 (4.43)	0.213
Cortisol awakening response (nmol/l time)	123	0.64 (1.29)	0.69 (1.41)	0.60 (1.18)	0.663
Total daily cortisol output (nmol/l time)	123	83.43 (65.38)	75.53 (46.05)	90.96 (79.22)	0.298
Cortisol AM/PM ratio	124	8.45 (7.27)	9.55 (8.71)	7.39 (5.39)	0.376
**Sleep (mean, SD)**^b^					
Sleep disturbances					
Total insomnia [0–15]	124	6.67 (3.51)	6.61 (2.86)	6.72 (3.99)	0.856
Sleep quality [0–15]	127	6.79 (3.87)	7.19 (3.39)	6.46 (4.21)	0.255
Sleep apnea [0–10]	114	1.46 (2.30)	1.25 (1.92)	1.64 (2.58)	0.510
Daytime sleepiness (during work time) [0–10]	118	2.86 (2.51)	2.83 (2.31)	2.89 (2.69)	0.842
Global sleep score [0–90]	93	28.84 (14.30)	30.69 (10.04)	26.76 (16.93)	0.113
Initial insomnia [0–5]	131	2.01 (1.49)	1.88 (1.28)	2.11 (1.64)	0.580
Middle insomnia [0–5]	129	2.26 (1.48)	2.22 (1.31)	2.28 (1.61)	0.975
Terminal insomnia [0–5]	126	2.30 (1.44)	2.43 (1.26)	2.19 (1.58)	0.290
Insufficient sleep [0–5]	128	2.01 (1.41)	2.07 (1.24)	1.96 (1.54)	0.535
Difficulty waking up [0–5]	130	1.32 (1.45)	1.50 (1.42)	1.17 (1.47)	0.089
Nightmares [0–5]	129	1.29 (1.22)	1.05 (1.01)	1.40 (1.36)	0.209
Restless leg [0–5]	128	1.02 (1.30)	0.88 (1.10)	1.13 (1.45)	0.673
Snoring [0–5]	124	1.52 (1.55)	1.43 (1.34)	1.61 (1.72)	0.971
General sleep [0–4]	130	1.83 (1.14)	1.82 (1.02)	1.84 (1.24)	0.923
Sleep indexes					
Daytime sleepiness [0–15]	126	5.30 (3.66)	5.41 (3.32)	5.21 (3.94)	0.603
Sleep quality [0–20]	123	8.92 (4.65)	8.98 (3.90)	8.87 (5.21)	0.890
Non-restorative sleep [0–15]	127	5.87 (3.82)	6.34 (3.49)	5.46 (4.06)	0.115
Sleep apnea [0–15]	114	2.84 (3.29)	2.55 (2.77)	3.10 (3.68)	0.711

Group comparisons are analyzed with a=chi-squared test (sex, smoking behavior and sleep medication), b= Wilcoxon rank sum test (age, depressive symptoms, AD biomarkers, imaging score, cortisol measures, and sleep measures except sleep quality index), c= t-test for education and sleep quality index, and d=fisher exact test (alcohol consumption). MTA = medial temporal atrophy (Scheltens scale), GCA = Global Cortical Atrophy (Pasquier scale), PA = Posterior atrophy (Koedam scale), WMH = White-matter Hyperintensities (Fazekas scale). Depressive symptoms are evaluated with GDS-15 (Geriatric Depression Scale).

### Associations between sleep disturbances and visual rating scales

Each sleep variable (Table 1) was modeled against each visual rating scale using ordinal logistic regression. Results for all sleep indexes are presented in Table 3, see supplementary S1 for analysis of all sleep disturbances parameters. Regression analysis showed that increased sleep apnea index was associated with significantly higher odds of increased posterior brain atrophy measured by the Koedam scale (OR = 1.20, 95% CI = [1.04 – 1.39]). No sex differences were observed (Females: OR = 1.25, p = 0.095, Males: OR = 1.14, p = 0.180). Following the addition of depressive symptoms and sleep medication as covariates to the models, this association remained significant (OR = 1.24, p = 0.007). All other associations were not significant (p>0.05, see Table 3 and S1).

**Table 3:** Associations between sleep difficulties and structural brain measures

	MTA (N=143)			GCA (N=140)			PA (N=73)			WMH (N=133)		
	OR	95% CI	p	OR	95% CI	p	OR	95% CI	p	OR	95% CI	p
Sleep indexes												
Daytime sleepiness	0.93	0.85 – 1.02	0.108	1.02	0.93 – 1.12	0.601	0.99	0.83 – 1.16	0.861	1.03	0.93 – 1.13	0.598
Sleep quality	0.99	0.92 – 1.06	0.717	1.01	0.93 – 1.09	0.864	1.00	0.87 – 1.13	0.945	1.03	0.95 – 1.11	0.522
Non-restorative sleep	0.96	0.87 – 1.05	0.324	0.95	0.87 – 1.05	0.321	0.92	0.79 – 1.08	0.323	1.02	0.93 – 1.12	0.669
Sleep apnea	0.95	0.85 – 1.05	0.332	0.96	0.85 – 1.08	0.510	1.20	1.04 – 1.39	0.015	0.97	0.85 – 1.11	0.673

Ordered logistic regression models controlled for age, sex, education, and alcohol consumption. MTA = medial temporal atrophy (Scheltens scale), GCA = Global Cortical Atrophy (Pasquier scale), PA = Posterior atrophy (Koedam scale), WMH = White-matter Hyperintensities (Fazekas scale).

### Associations between sleep disturbances and diurnal cortisol levels

Linear regressions showed some significant associations between sleep indexes and cortisol levels (Table 4). See supplementary S2 for regression analysis of all sleep disturbance parameters. Higher sleepiness index (β = −0.03; 95% CI = [−0.06 – 0.00]) and increased sleep apnea index (β = −0.03; 95% CI = [−0.07 – 0.00]) were both associated with reduced awakening cortisol. The association between sleepiness index and awakening cortisol was only significant in females [Females: β = -0.05, p = 0.025, Males: β = −0.01, p = 0.598], whereas the association between apnea index and awakening cortisol was non-significant in both sexes, although closer to significance for males [Females: β = −0.02, p = 0.428, Males: β = −0.04, p = 0.061]. Increased sleepiness index was also associated with a reduced AM/PM cortisol ratio (β = −0.04; 95% CI = [−0.08 – −0.01]), reflecting a flatter diurnal cortisol pattern. No sex difference was found when stratifying [Females: β = −0.04, p = 0.136, Males: β = −0.03, p = 0.410]. Following the addition of depressive symptoms and sleep medication as covariates to the models, both associations with awakening cortisol remained significant [Sleepiness index: β = −0.03, p = 0.045, Apnea index = β = −0.04, p = 0.045], while the association with AM/PM cortisol ratio remained negative but was slightly attenuated [β = −0.04, p=0.055]. None of the sleep items showed significant associations with bedtime cortisol, CAR, or total daily cortisol output (p>0.05, see Table 4 and S2).

**Table 4:** Associations between sleep disturbances and computed cortisol levels.

	Awakening cortisol			Bedtime cortisol			CAR			Total cortisol			Cortisol AM/PM ratio		
	β	95% CI	p	β	95% CI	p	β	95% CI	p	β	95% CI	p	β	95% CI	p
Sleep indexes															
Daytime sleepiness	−0.03	−0.06 – 0.00	0.025	0.02	−0.03 – 0.07	0.479	0.00	−0.07 – 0.07	0.998	−0.01	−0.04 – 0.03	0.682	−0.04	−0.08 – −0.01	0.021
Sleep quality	−0.01	−0.04 – 0.01	0.304	0.02	−0.02 – 0.07	0.296	−0.01	−0.07 – 0.04	0.657	−0.01	−0.04 – 0.02	0.537	−0.03	−0.06 – 0.01	0.106
Non-restorative sleep	−0.03	−0.05 – 0.00	0.074	0.02	−0.03 – 0.07	0.524	−0.01	−0.07 – 0.05	0.766	−0.01	−0.04 – 0.03	0.662	−0.03	−0.07 – 0.01	0.090
Sleep apnea	−0.03	−0.07 – 0.00	0.045	−0.02	−0.08 – 0.05	0.599	−0.01	−0.09 – 0.07	0.794	−0.02	−0.06 – 0.03	0.460	−0.01	−0.06 – 0.03	0.521

Linear regression models controlled for age, sex, education, and alcohol consumption. CAR = Cortisol awakening response.

### Interactions between sleep and cortisol when predicting structural brain measures

The significant association between the KSQ sleep apnea index and posterior atrophy (Table 3) was further analyzed with cortisol as an interaction term, to investigate a potential interaction between sleep and cortisol for predicting neuroimaging correlates. The association did not have a significant interaction with cortisol (p>0.05).

## Discussion

This study revealed associations between distinct self-reported sleep parameters with increased PA and altered diurnal cortisol patterns among memory clinic participants. The sleep apnea index was associated with higher odds of PA, and the sleepiness and sleep apnea index were both associated with reduced awakening cortisol, and sleepiness was further associated with a lower AM/PM cortisol ratio. No other investigated associations were significant.

Our results showed that increased sleep apnea index is associated with greater odds of PA. Sleep apnea was expected to be associated with neuroimaging correlates in a memory clinic cohort, as objective measures of SDB are associated with earlier onset of cognitive decline (54), and widespread structural brain changes (22). For older adults at-risk of dementia, two OSA severity components were associated with widespread structural brain correlates; OSA oxygen desaturation was associated with reduced thickness of the temporal lobe, whereas OSA related sleep disturbances were associated with increased thickness in the postcentral gyrus and pericalcarine (22). Our KSQ sleep apnea index and their OSA related sleep disturbances are both associated with pathology in the parietal lobe. However, our results of PA may reflect more sulci widening, whereas previous study results (22) reflect hypertrophy. Together with previous results, this suggests that apnea results in widespread brain changes, which might be explained by different apnea components affecting the brain differently (22). Furthermore, apnea severity and duration are also suggested to affect the neuroimaging outcomes (55).

Intermittent hypoxia and reoxygenation occurring in apnea leads to several maladaptive processes, such as increased activation of astroglia and microglia, increased glia proliferation, increased phosphorylated and total tau, and amyloid β (55). Regarding AD, which is characterized by amyloid genesis and tau phosphorylation, these latter maladaptive processes become particularly interesting. This implies that sleep apnea could possibly be associated with increased AD risk though such pathologies. While we did not find associations between sleep apnea index and MTA, dementia with posterior cortical atrophy (PCA) is considered a posteriorly shifted variant of AD (56). Therefore, sleep apnea and its association with increased odds of PA, resembles the pathology seen in PCA.

Furthermore, in OSA there are changes in both macro-and micro-level sleep architecture (55). People with OSA tend to have altered proportions of sleep stages, including an increased proportion of N2 stage, and less N1, N3 and REM sleep (55). Microlevel sleep architecture changes are also observed, as there is a decay of slow-wave sleep activity upon even mild OSA (55). Therefore, there are widespread effects happening upon sleep apnea that potentially could explain the underlying association between increased KSQ sleep apnea index and increased odds of PA. However, with the current data it is not possible to examine the underlying mechanisms.

OSA is a highly prevalent condition that has strong associations with older age (55). As treatment options for OSA do exist, it is important to understand the association with neurodegenerative pathology and AD, such that we potentially can prevent or delay further pathology and AD incidence.

No significant associations were found between sleep apnea and MTA, GCA or WM, and no other sleep difficulties were found to associate with any of the 4 visual rating scales. A previous meta-analysis showed that, besides SDB, a variety of sleep disturbances (insomnia, excessive daytime sleepiness, sleep-related movement disorder, sleep pattern change, and nonspecific sleep problems) are associated with an increased risk of developing dementia in older adults (2). When studying the association between sleep disturbances and neuroimaging correlates, several different associations have been found. Insomnia was associated with reduced hippocampal volume (9), and reduced GM in areas of the frontal (12, 18) and parietal lobe (12, 17), in individuals living with insomnia. This study did not find such relationships for MTA, GCA, nor PA, which could have resembled neuroimaging outcomes found in previous studies. This could indicate that sleep disturbances resembling insomnia must be more severe to show associations with structural brain changes visible on visual rating scales. A previous study in participants without a sleep diagnosis found that more self-reported early awakenings, a symptom of terminal insomnia, were associated with reduced GM density in the frontal lobe (57). Therefore, the differences in findings could also partly be explained by the difference in cohort characteristics as previous studies investigated cognitively heathy adults (mean age ∼60 years (12), ∼56 years (17), ∼40 years (18, 57)), which is in contrast to our memory clinic cohort (mean age 61 years). Sleep duration has also been previously associated with structural brain changes, as self-reported short sleep (<6h (58) and <7h (59)) was associated with increased WMH in middle-aged adults (58) and in young adults with cortical thinning (59), in cognitively healthy participants. Poor sleep quality, based on the Pittsburgh Sleep Quality Index (PSQI), has been associated with decreased GM in frontal cortex (60) and increased GCA (61), in cognitively healthy adults (age: 20 – 84 (60); age: 61–95 (61)). Lastly, self-reported measures of excessive daytime sleepiness were associated with cortical thinning and reduced hippocampal volume in cognitively healthy older adults (11).

With our cohort and methods, we did not find significant associations between sleep disturbances, other than apnea symptoms, and structural brain measures. However, when comparing our study to previous studies investigating the association between sleep disturbances and brain imaging correlates, this study cohort differs as it includes memory clinic participants with either SCI or MCI, compared to cognitively healthy participants. Further, this and previous studies differ in how structural brain measures are quantified. All previous studies, except one (61), used volumetric measures to quantify structural brain measures. By contrast, this clinical study utilized visual rating scales, which is a more accessible approach to quantify structural brain measures in clinical settings (62). There are several studies that report good comparison between visual rating scales and MRI segmentation upon AD diagnosis (62, 63). However, it may be that the visual rating scales are not sensitive to the mechanistic changes happening due to sleep disturbances. Lastly, this study examines the association of neuroimaging correlates with sleep disturbances that have not reached a severity of a sleep disorder. It may be that the duration and severity of sleep disturbances are important in their potential associations with brain imaging correlates.

### Sleep disturbances and diurnal cortisol patterns

The memory clinic participants who were sleepier during daytime and those who reported more sleep apnea symptoms, had reduced awakening cortisol levels. This could be explained by participants having lighter or more disrupted sleep, meaning that at awakening the cortisol had already been elevated for a while, such that at the measurement time cortisol was already in the declining phase. The association for sleepiness index was seen to be driven by females, as only female participants showed significant association between sleepiness index and awakening cortisol upon stratification analysis. Further, the association for apnea index was stronger in males compared to females, which may derive from the fact that sleep apnea has a higher prevalence in males than females (53). Secondly, the memory clinic participants who reported higher daytime sleepiness had a significantly reduced AM/PM cortisol ratio, reflecting a flattened diurnal cortisol pattern. Aging may be associated with a reduced variation in the diurnal cortisol rhythm, and aging accompanied by AD is seen to have an even greater reduction in the amplitude of the diurnal cortisol rhythm (25). It is reported that a flattening of the cortisol rhythm might be secondary to the changes in receptor activity in the hippocampus, as specific receptors (mineralocorticoid receptors) are the target of a negative feedback loop for cortisol (25). Thus, seeing a flatter daily cortisol rhythm upon higher daytime sleepiness could indicate that poor sleep is associated with an unhealthy diurnal cortisol profile, similar to what is observed among individuals with AD (25). However, besides hippocampal atrophy, a flatter diurnal cortisol profile could also be explained by increased napping, which is known to increase with aging (64). As, we do not have any data on napping, thus this could not be examined further.

We found increased daytime sleepiness and apnea to be associated with reduced awakening cortisol. This is in line with a previous study by Backhaus et al. who found that insomnia patients have reduced awakening cortisol (saliva) compared to healthy sleepers (37), and that, for both patients and controls, low awakening cortisol was associated with a reduced “feeling of recovery” after sleep (37). Such associations between reduced awakening cortisol and a lower “feeling of recovery”, are similar to our results showing associations between reduced awakening cortisol and increased daytime sleepiness. These consistent results are found despite differences in cohort characteristics, as Backhaus et al. studied cognitively healthy insomnia participants in the age range 32–61 years (37). On the other hand, Basta et al. found that within a group of participants with MCI, those who had insomnia and short sleep, had higher morning cortisol (blood), compared to participants with MCI without sleep disturbances (34). This study is in line with the theory of sleep disturbances being linked with HPA-axis hyperactivation (28). In the study by Basta et al. blood samples were collected between 10:00 AM and 12:00 PM, whereas this study uses saliva samples collected immediately after awakening. The diurnal cortisol pattern varies greatly in the morning (50), and thus the difference in sampling time and source may explain the different associations found.

We did not find any association between sleep measures and evening cortisol, even though a previous study has found that male insomnia patients (mean age 40 years) had increased evening cortisol compared to healthy sleepers (30). This inconsistency may be due to differences in the study populations, including age, sex, cognitive status, and presence of insomnia diagnosis. Furthermore, this study did not find any relation between self-reported sleep parameters and total daily cortisol levels, which is in line with previous studies that only found such associations when using objective sleep measures (29, 32). Lastly, no interactions were found between cortisol and sleep, upon associations with neuroimaging correlates. This cannot be compared with previous studies, as this study, to our knowledge, is the first to perform such analyses in a memory clinic cohort.

### Limitations and strengths of the study

First, this study has, due to its sample size and exploratory nature, not performed corrections for multiple comparisons, and therefore all associations should be interpreted with caution. Second, this is a cross-sectional study which is unable to infer the directionality between sleep and structural brain changes, and between sleep and cortisol dysregulation. Third, this study used visual rating scales for quantifying brain measures. Further the visual rating scales are based on either CT or MRI. The sample size of this cohort did not allow for separate analysis of MRI and CT. Previous studies (11, 57–59) indicate the value of performing volumetric MRI measures when investigating the associations between structural brain measures and sleep disturbances. Visual rating scales represent simpler methods, but they also have their strengths as they are more applicable in clinical settings (43). However, future studies would benefit from controlling for imaging modality. Fourth, this study uses self-reported sleep, in contrast to objective sleep measures. By using questionnaires to quantify sleep disturbances we might not capture all sleep disturbances when studying participants with either SCI or MCI, potentially masking possible associations between sleep disturbances, structural brain measures and cortisol in a memory clinic cohort. There is still not a well characterized relationship between self-reported and objective sleep (65), and these measures may reflect different underlying biological and physiological outcomes (66). However, previous studies (29, 32) have highlighted the necessity to use objective measures when investigating associations between sleep and cortisol levels. Also, sleep studies measures sleep using a variety of questionnaires (1, 65), which further complicates comparisons of results. Fifth, due to sample size and data availability, none of the analyses have controlled for cardiometabolic risk factors, even though these are known to have great impact on sleep (67). Further research is needed to identify the role of cardiometabolic factors in these associations. Sixth, this study combines participants with SCI and MCI without additional analyses to account for AD biomarkers. With the exploratory nature of this study, it did not have the power to control or stratify for amyloid pathology. However future studies with larger sample sizes would benefit from including AD biomarkers and/or cognitive status in the regression models. Lastly, this study did not have data on napping, which could have an influence on the diurnal cortisol profile. On the other hand, this study has several strengths as it provides important insights to understanding the role of sleep disturbances in adults with cognitive impairment, a topic that is timely and clinically relevant. Firstly, the study included multiple validated measures of structural brain measures, and all are well implemented in the clinic. Secondly, the study included high temporal sampling of saliva, allowing us to measure the diurnal cortisol profile using several indicators. Thirdly, the study measures overall sleep problems, in comparison to focusing on specific sleep diagnoses, which is in line with research showing that various sleep disturbances are associated with higher risk of AD (2, 68). Fourth, the study investigates sex differences, which are important considering both the sex differences in the prevalence of dementia and AD, as well as the sex differences in sleep disturbances. Further, this study is based on a memory clinic cohort without sleep diagnosis, enabling us to investigate participants with some cognitive impairment who have subclinical sleep disturbances. Sleep studies on memory clinic participants are sparse, and thus this study sheds light on sleep in this population.

This study holds great relevance for clinical practice and dementia prevention. Assessments of certain sleep parameters in memory clinic patients may help in identifying risk factors that contribute to cognitive impairment and can promote the inclusion of sleep interventions and treatments to prevent further cognitive decline. Further, this study holds great importance for dementia prevention initiatives, as it adds to our understanding of the role of sleep as an emerging risk factor (1). The first multidomain interventions for dementia, the FINGER study (69), did not include sleep as an intervention, whereas later FINGER-based studies, such as the Netherlands FINGER (FINGER-NL) study (70) have included sleep counseling as an additional intervention component. This study further strengthens the evidence on the importance of sleep for cognitive health among older adults, and especially among individuals with subjective impairment or MCI.

## Conclusion

In conclusion, this study shows that increased self-reported sleep apnea symptoms are associated with neuroimaging correlates of posterior atrophy, and that increased daytime sleepiness and apnea symptoms are associated with cortisol dysregulation, in memory clinic participants. Sleep is considered an emerging risk factor for dementia (1), however further evidence is needed on the underlying mechanisms. This study contributes to knowledge about which sleep parameters are associated with MRI and cortisol measures among individuals with subjective and mild cognitive impairment. However, future longitudinal studies with a larger number of participants using volumetric measures for quantifying structural brain measures and sleep disturbances, are needed to further understand the underlying mechanisms. Furthermore, additional research is needed to better understand the factors underlying the sex differences observed.

## Supplementary material

Supplementary material, approximately 27.2 KB.

## Data Availability

*Data Availability statement:* The research team is open to requests for data collected in this study. Study plan (including the research question, planned analysis, and data required) will be evaluated on a case-by-case basis. Shared data will encompass the data dictionary and de-identified data only. Analysis will be conducted in collaboration with the research team. Access is subject to the GEDOC legal framework. An access agreement will be prepared.

## Notes

*Ethical standards:* The study was approved by the Regional Ethical Review Board (Stockholm 2014/524-31/1). All participants gave informed consent to participate in the study.

## References

[1] Livingston G, Huntley J, Sommerlad A, et al (2020) Dementia prevention, intervention, and care: 2020 report of the Lancet Commission. Lancet 396:413–446 doi:10.1016/S0140-6736(20)30367-6 32738937 10.1016/S0140-6736(20)30367-6 PMC7392084

[2] Shi L, Chen SJ, Ma MY, Bao YP, Han Y, Wang YM, Shi J, Vitiello M V., Lu L (2018) Sleep disturbances increase the risk of dementia: A systematic review and meta-analysis. Sleep Med Rev 40:4–16 doi:10.1016/j.smrv.2017.06.010 28890168 10.1016/j.smrv.2017.06.010

[3] Brenowitz WD, Xiang Y, Mcevoy CT, Yang C, Yaffe K, Le W-D, Leng Y (2021) Current Alzheimer disease research highlights: evidence for novel risk factors. 10.1097/CM9.0000000000001706 doi:10.1097/CM9.0000000000001706 10.1097/CM9.0000000000001706 PMC8478399 34507318

[4] Grigg-Damberger M, Foldvary-Schaefer N (2022) Sleep Biomarkers Help Predict the Development of Alzheimer Disease. J Clin Neurophysiol Publish Ah:1-8 doi:10.1097/wnp.0000000000000818 10.1097/WNP.0000000000000818 35239558

[5] Miner B, Kryger MH (2017) Sleep in the aging population. Sleep Med Clin 176:139–148 doi:10.1016/j.jsmc.2016.10.008.Sleep 10.1016/j.jsmc.2016.10.008 PMC5300306 28159095

[6] Sexton CE, Sykara K, Karageorgiou E, Zitser J, Rosa T, Yaffe K, Leng Y (2020) Connections Between Insomnia and Cognitive Aging. Neurosci Bull 36:77–84 doi:10.1007/s12264-019-00401-9 31222500 10.1007/s12264-019-00401-9 PMC6940406

[7] Palagini L, Hertenstein E, Riemann D, Nissen C (2022) Sleep, insomnia and mental health. J Sleep Res. 10.1111/JSR.13628 doi:10.1111/JSR.13628 10.1111/jsr.13628 35506356

[8] Brodt S, Inostroza M, Niethard N, Born J (2023) Review Sleep — A brain-state serving systems memory consolidation. Neuron 111:1050–1075 doi:10.1016/j.neuron.2023.03.005 37023710 10.1016/j.neuron.2023.03.005

[9] Riemann D, Voderholzer U, Spiegelhalder K, Hornyak M, Buysse DJ, Nissen C, Hennig J, Perlis ML, Van Elst LT, Feige B (2007) Chronic insomnia and MRI-measured hippocampal volumes: A pilot study. Sleep 30:955–958 doi:10.1093/sleep/30.8.955 17702263 10.1093/sleep/30.8.955 PMC1978381

[10] Joo EY, Kim H, Suh S, Hong SB (2014) Hippocampal substructural vulnerability to sleep disturbance and cognitive impairment in patients with chronic primary insomnia: Magnetic resonance imaging morphometry. Sleep 37:1189–1198 doi:10.5665/sleep.3836 25061247 10.5665/sleep.3836 PMC4098804

[11] Carvalho DZ, St. Louis EK, Boeve BF, et al (2017) Excessive daytime sleepiness and fatigue may indicate accelerated brain aging in cognitively normal late middle-aged and older adults. Sleep Med 32:236–243 doi:10.1016/J.SLEEP.2016.08.023 28065685 10.1016/j.sleep.2016.08.023 PMC5551490

[12] Altena E, Vrenken H, Van Der Werf YD, van den Heuvel OA, Van Someren EJW (2010) Reduced Orbitofrontal and Parietal Gray Matter in Chronic Insomnia: A Voxel-Based Morphometric Study. Biol Psychiatry 67:182–185 doi:10.1016/J.BIOPSYCH.2009.08.003 19782344 10.1016/j.biopsych.2009.08.003

[13] Noh HJ, Joo EY, Kim ST, Yoon SM, Koo DL, Kim D, Lee GH, Hong SB (2012) The relationship between hippocampal volume and cognition in patients with chronic primary insomnia. J Clin Neurol 8:130–138 doi:10.3988/jcn.2012.8.2.130 22787497 10.3988/jcn.2012.8.2.130 PMC3391618

[14] Spiegelhalder K, Regen W, Baglioni C, Klöppel S, Abdulkadir A, Hennig J, Nissen C, Riemann D, Feige B (2013) Insomnia does not appear to be associated with substantial structural brain changes. Sleep 36:731–737 doi:10.5665/sleep.2638 23633756 10.5665/sleep.2638 PMC3624828

[15] Winkelman JW, Benson KL, Buxton OM, Lyoo IK, Yoon S, O’Connor S, Renshaw PF (2010) Lack of hippocampal volume differences in primary insomnia and good sleeper controls: An MRI volumetric study at 3 Tesla. Sleep Med 11:576–582 doi:10.1016/J.SLEEP.2010.03.009 20466585 10.1016/j.sleep.2010.03.009

[16] Winkelman JW, Plante DT, Schoerning L, Benson K, Buxton OM, O’Connor S, Jensen JE, Renshaw PF, Gonenc A (2013) Increased Rostral Anterior Cingulate Cortex Volume in Chronic Primary Insomnia. Sleep 36:991–998 doi:10.5665/sleep.2794 23814335 10.5665/sleep.2794 PMC3669070

[17] Grau-Rivera O, Operto G, Falcon C, et al (2020) Association between insomnia and cognitive performance, gray matter volume, and white matter microstructure in cognitively unimpaired adults. Alzheimer’s Dement 15:P207–P209 doi:10.1016/j.jalz.2019.06.4549 10.1186/s13195-019-0547-3 PMC6945611 31907066

[18] Li M, Yan J, Li S, Wang T, Wen H, Yin Y, Fu S, Zeng L, Tian J, Jiang G (2018) Altered gray matter volume in primary insomnia patients: a DARTEL-VBM study. Brain Imaging Behav 12:1759–1767 doi:10.1007/s11682-018-9844-x 29411240 10.1007/s11682-018-9844-x

[19] Joo EY, Noh hyun jin, Kim J-S, Koo DL, Kin D, Hwang KJ, Kim JY, Kim ST, Kim MR, Hong SB (2013) Brain Gray Matter Deficits in Patients with Chronic Primary Insomniae. Sleep 36:999–1007 doi:10.5665/sleep.2796 23814336 10.5665/sleep.2796 PMC3669067

[20] Yu S, Feng F, Zhang Q, Shen Z, Wang Z, Hu Y, Gong L (2020) Gray matter hypertrophy in primary insomnia: a surface-based morphometric study. Brain Imaging Behav 14:1309–1317 doi:10.1007/s11682-018-9992-z 30511119 10.1007/s11682-018-9992-z

[21] Baril AA, Gagnon K, Descoteaux M, et al (2020) Cerebral white matter diffusion properties and free-water with obstructive sleep apnea severity in older adults. Hum Brain Mapp 41:2686–2701 doi:10.1002/hbm.24971 32166865 10.1002/hbm.24971 PMC7294053

[22] Cross NE, Memarian N, Duffy SL, Paquola C, LaMonica H, D’Rozario A, Lewis SJG, Hickie IB, Grunstein RR, Naismith SL (2018) Structural brain correlates of obstructive sleep apnoea in older adults at risk for dementia. Eur Respir J. 10.1183/13993003.00740-2018 doi:10.1183/13993003.00740-2018 10.1183/13993003.00740-2018 29973356

[23] Morrell MJ, Jackson ML, Twigg GL, et al (2010) Changes in brain morphology in patients with obstructive sleep apnoea. Thorax 65:908–914 doi:10.1136/thx.2009.126730 20861295 10.1136/thx.2009.126730

[24] Alperin N, Wiltshire J, Lee SH, Ramos AR, Hernandez-Cardenache R, Rundek T, Curiel Cid R, Loewenstein D (2019) Effect of sleep quality on amnestic mild cognitive impairment vulnerable brain regions in cognitively normal elderly individuals. Sleep 42:1–10 doi:10.1093/sleep/zsy254 10.1093/sleep/zsy254 PMC6424074 30541112

[25] Buckley TM, Schatzberg AF (2005) Aging and the role of the HPA axis and rhythm in sleep and memory-consolidation. Am J Geriatr Psychiatry 13:344–352 doi:10.1097/00019442-200505000-00002 15879582 10.1176/appi.ajgp.13.5.344

[26] van Dalfsen JH, Markus CR (2018) The influence of sleep on human hypothalamic-pituitary-adrenal (HPA) axis reactivity: A systematic review. Sleep Med Rev 39:187–194 doi:10.1016/j.smrv.2017.10.002 29126903 10.1016/j.smrv.2017.10.002

[27] Dressle RJ, Feige B, Spiegelhalder K, Schmucker C, Benz F, Mey NC, Riemann D (2022) HPA axis activity in patients with chronic insomnia: A systematic review and meta-analysis of case-control studies. Sleep Med Rev 62:101588 doi:10.1016/j.smrv.2022.101588 35091194 10.1016/j.smrv.2022.101588

[28] Van Cauter E, Balbo M, Leproult R (2010) Impact of sleep and its disturbances on hypothalamo-pituitary-adrenal axis activity. Int J Endocrinol. 10.1155/2010/759234 doi:10.1155/2010/759234 10.1155/2010/759234 PMC2902103 20628523

[29] Grimaldi D, Reid KJ, Papalambros NA, Braun RI, Malkani RG, Abbott SM, Ong JC, Zee PC (2021) Autonomic dysregulation and sleep homeostasis in insomnia. Sleep 44:1–13 doi:10.1093/sleep/zsaa274 10.1093/sleep/zsaa274 PMC8343579 33295989

[30] Rodenbeck A, Huether G, Rüther E, Hajak G (2002) Interactions between evening and nocturnal cortisol secretion and sleep parameters in patients with severe chronic primary insomnia. Neurosci Lett 324:159–163 doi:10.1016/S0304-3940(02)00192-1 11988351 10.1016/s0304-3940(02)00192-1

[31] D’Aurea CVR, Poyares D, Piovezan RD, Passos G, Tufik S, de Mello MT (2015) Objective short sleep duration is associated with the activity of the hypothalamic-pituitary-adrenal axis in insomnia. Arq Neuropsiquiatr 73:516–519 doi:10.1590/0004-282X20150053 26083888 10.1590/0004-282X20150053

[32] Morgan E, Schumm LP, McClintock M, Waite L, Lauderdale DS (2017) Sleep characteristics and daytime cortisol levels in older adults. Sleep. 10.1093/sleep/zsx043 doi:10.1093/sleep/zsx043 10.1093/sleep/zsx043 PMC6251667 28329370

[33] Dronse J, Ohndorf A, Richter N, et al (2023) Serum cortisol is negatively related to hippocampal volume, brain structure, and memory performance in healthy aging and Alzheimer’s disease. Front Aging Neurosci 15:1–14 doi:10.3389/fnagi.2023.1154112 10.3389/fnagi.2023.1154112 PMC10213232 37251803

[34] Basta M, Vgontzas AN, Fernandez-Mendoza J, Antypa D, Li Y, Zaganas I, Panagiotakis S, Karagkouni E, Simos P (2022) Basal Cortisol Levels Are Increased in Patients with Mild Cognitive Impairment: Role of Insomnia and Short Sleep Duration. J Alzheimer’s Dis 87:933–944 doi:10.3233/jad-215523 35404277 10.3233/JAD-215523

[35] Backhaus J, Junghanns K, Born J, Hohaus K, Faasch F, Hohagen F (2006) Impaired Declarative Memory Consolidation During Sleep in Patients With Primary Insomnia: Influence of Sleep Architecture and Nocturnal Cortisol Release. Biol Psychiatry 60:1324–1330 doi:10.1016/j.biopsych.2006.03.051 16876140 10.1016/j.biopsych.2006.03.051

[36] Riemann D, Klein T, Rodenbeck A, Feige B, Horny A, Hummel R, Weske G, Al-Shajlawi A, Voderholzer U (2002) Nocturnal cortisol and melatonin secretion in primary insomnia. Psychiatry Res 113:17–27 doi:10.1016/S0165-1781(02)00249-4 12467942 10.1016/s0165-1781(02)00249-4

[37] Backhaus J, Junghanns K, Hohagen F (2004) Sleep disturbances are correlated with decreased morning awakening salivary cortisol. Psychoneuroendocrinology 29:1184–1191 doi:10.1016/j.psyneuen.2004.01.010 15219642 10.1016/j.psyneuen.2004.01.010

[38] Holleman J, Adagunodo S, Kåreholt I, Hagman G, Aspö M, Udeh-Momoh CT, Solomon A, Kivipelto M, Sindi S (2022) Cortisol, cognition and Alzheimer’s disease biomarkers among memory clinic patients. BMJ Neurol Open 4:e000344 doi:10.1136/bmjno-2022-000344 36277478 10.1136/bmjno-2022-000344 PMC9582323

[39] Geneva; WHOISC of D and RHP-TR World Health Organization. International Statistical Classification of Disease and Related Health Problems - Thenth Revision. Geneva; 1992.

[40] Winblad B, Palmer K, Kivipelto M, et al (2004) Mild cognitive impairment - Beyond controversies, towards a consensus: Report of the International Working Group on Mild Cognitive Impairment. J Intern Med 256:240–246 doi:10.1111/j.1365-2796.2004.01380.x 15324367 10.1111/j.1365-2796.2004.01380.x

[41] Jessen F, Amariglio RE, Buckley RF, et al (2020) The characterisation of subjective cognitive decline. Lancet Neurol 19:271–278 doi:10.1016/S1474-4422(19)30368-0 31958406 10.1016/S1474-4422(19)30368-0 PMC7062546

[42] Nordin M, Åkerstedt T, Nordin S (2013) Psychometric evaluation and normative data for the karolinska sleep questionnaire. Sleep Biol Rhythms 11:216–226 doi:10.1111/sbr.12024

[43] Harper L, Barkhof F, Fox NC, Schott JM (2015) Using visual rating to diagnose dementia: A critical evaluation of MRI atrophy scales. J Neurol Neurosurg Psychiatry 86:1225–1233 doi:10.1136/jnnp-2014-310090 25872513 10.1136/jnnp-2014-310090

[44] Wahlund LO, Westman E, van Westen D, Wallin A, Shams S, Cavallin L, Larsson EM (2017) Imaging biomarkers of dementia: recommended visual rating scales with teaching cases. Insights Imaging 8:79–90 doi:10.1007/s13244-016-0521-6 28004274 10.1007/s13244-016-0521-6 PMC5265189

[45] Scheltens P, Leys D, Barkhof F, Huglo D, Weinstein H, Vermersch P, Kuiper M, Steinling M, Wolters EC, Walk J (1992) Atrophy of medial temporal lobes on MRI in “probable” Alzheimer’s disease and normal ageing: diagnostic value and normal againg: diagnostic value and neuropsychological correlates. J Neurol Neurosurg Psychiatry 55:967–972 1431963 10.1136/jnnp.55.10.967 PMC1015202

[46] Florence P, Didier L, G.E. WJ, Francois M-V, Frederik B, Scheltens Philip (1996) Inter- and Intraobserver Reproducibility of Cerebral Atrophy Assessment on MRI Scans with Hemispheric Infarcts. Eur Neurol 36:268–272 8864706 10.1159/000117270

[47] Koedam ELGE, Lehmann M, Van Der Flier WM, Scheltens P, Pijnenburg YAL, Fox N, Barkhof F, Wattjes MP (2011) Visual assessment of posterior atrophy development of a MRI rating scale. Eur Radiol 21:2618–2625 doi:10.1007/s00330-011-2205-4 21805370 10.1007/s00330-011-2205-4 PMC3217148

[48] Fazekas F, Chawluk JB, Alavi A (1987) MR signal abnormalities at 1.5 T in Alzheimer’s dementia and normal aging. Am J Neuroradiol 8:421–426 10.2214/ajr.149.2.351 3496763

[49] Ct R, Wattjes MP, Henneman WJP, Tra F (2009) Diagnostic Imaging of Patients in a Memory Clinic: Comparison of MR Imaging and 64 - Detector Purpose: Methods: Results: Conclusion: 253 10.1148/radiol.2531082262 19635835

[50] Adam EK, Kumari M (2009) Assessing salivary cortisol in large-scale, epidemiological research. Psychoneuroendocrinology 34:1423–1436 doi:10.1016/j.psyneuen.2009.06.011 19647372 10.1016/j.psyneuen.2009.06.011

[51] Pruessner JC, Kirschbaum C, Meinlschmid G, Hellhammer DH (2003) Two formulas for computation of the area under the curve represent measures of total hormone concentration versus time-dependent change. Psychoneuroendocrinology 28:916–931 doi:10.1016/S0306-4530(02)00108-7 12892658 10.1016/s0306-4530(02)00108-7

[52] Mander BA, Winer JR, Walker MP (2017) Sleep and Human Aging. Neuron 94:19–36 doi:10.1016/J.NEURON.2017.02.004 28384471 10.1016/j.neuron.2017.02.004 PMC5810920

[53] Krishnan V, Collop NA (2006) Gender differences in sleep disorders. Curr Opin Pulm Med 12:383–389 doi:10.1097/01.mcp.0000245705.69440.6a 17053485 10.1097/01.mcp.0000245705.69440.6a

[54] Osorio RS, Gumb T, Pirraglia E, et al (2015) Sleep-disordered breathing advances cognitive decline in the elderly. Neurology 84:1964–1971 doi:10.1212/WNL.0000000000001566 25878183 10.1212/WNL.0000000000001566 PMC4433459

[55] Rosenzweig I, Glasser M, Polsek D, Leschziner GD, Williams SCR, Morrell MJ (2015) Sleep apnoea and the brain: A complex relationship. Lancet Respir Med 3:404–414 doi:10.1016/S2213-2600(15)00090-9 25887982 10.1016/S2213-2600(15)00090-9

[56] Mendez MF, Ghajarania M, Perryman KM (2002) Posterior cortical atrophy: Clinical characteristics and differences compared to Alzheimer’s disease. Dement Geriatr Cogn Disord 14:33–40 doi:10.1159/000058331 12053130 10.1159/000058331

[57] Stoffers D, Moens S, Benjamins J, van Tol MJ, Penninx BWJH, Veltman DJ, Van der Wee NJA, Van Someren EJW (2012) Orbitofrontal gray matter relates to early morning awakening: A neural correlate of insomnia complaints? Front Neurol JUN:1-7 doi:10.3389/fneur.2012.00105 10.3389/fneur.2012.00105 PMC3463899 23060850

[58] Yaffe K, Nasrallah I, Hoang TD, Lauderdale DS, Knutson KL, Carnethon MR, Launer LJ, Cora;, Lewis E, Sidney S (2016) Sleep Duration and White Matter Quality in Middle-Aged Adults. Sleep. 10.5665/sleep.6104 doi:10.5665/sleep.6104 10.5665/sleep.6104 PMC4989263 27397561

[59] Spira AP, Gonzalez CE, Venkatraman VK, Wu MN, Pacheco J, Simonsick EM, Ferrucci L, Resnick SM (2016) Sleep duration and subsequent cortical thinning in cognitively normal older adults. Sleep 39:1121–1128 doi:10.5665/sleep.5768 26951390 10.5665/sleep.5768 PMC4835311

[60] Sexton CE, Storsve AB, Walhovd KB, Johansen-Berg H, Fjell AM (2014) Poor sleep quality is associated with increased cortical atrophy in community-dwelling adults. Neurology 83:967–973 doi:10.1212/WNL.0000000000000774 25186857 10.1212/WNL.0000000000000774 PMC4162301

[61] Del Brutto OH, Mera RM, Zambrano M, Castillo PR (2016) The association between poor sleep quality and global cortical atrophy is related to age. Results from the Atahualpa Project. Sleep Sci 9:147–150 doi:10.1016/j.slsci.2016.06.004 28123651 10.1016/j.slsci.2016.06.004 PMC5241584

[62] Loreto F, Gontsarova A, Scott G, Patel N, Win Z, Carswell C, Perry R, Malhotra P (2023) Visual atrophy rating scales and amyloid PET status in an Alzheimer’s disease clinical cohort. 10.1002/acn3.51749 doi:10.1002/acn3.51749 10.1002/acn3.51749 PMC10109315 36872523

[63] Young H, Chae P, Park R, Hyun C, Woo S, Shim H, Joon S (2021) Diagnostic performance of the medial temporal lobe atrophy scale in patients with Alzheimer’s disease: a systematic review and meta - analysis European Society of Neuroradiology. Eur Radiol 9060–9072 doi:10.1007/s00330-021-08227-8 10.1007/s00330-021-08227-8 34510246

[64] Deantoni M, Reyt M, Baillet M, et al (2023) Napping and circadian sleep-wake regulation during healthy aging. Sleep 1–13 doi:10.1093/sleep/zsad287 10.1093/sleep/zsad287 37943833

[65] Cudney LE, Frey BN, McCabe RE, Green SM (2022) Investigating the relationship between objective measures of sleep and self-report sleep quality in healthy adults: A review. J Clin Sleep Med 18:927–936 doi:10.5664/jcsm.9708 34609276 10.5664/jcsm.9708 PMC8883085

[66] Jackowska M, Ronaldson A, Brown J, Steptoe A (2016) Biological and psychological correlates of self-reported and objective sleep measures. J Psychosom Res 84:52–55 doi:10.1016/j.jpsychores.2016.03.017 27095159 10.1016/j.jpsychores.2016.03.017

[67] Humer E, Pieh C, Brandmayr G (2020) Metabolomics in sleep, insomnia and sleep apnea. Int J Mol Sci 21:1–17 doi:10.3390/ijms21197244 10.3390/ijms21197244 PMC7583860 33008070

[68] Sindi S, Kåreholt I, Johansson L, et al (2018) Sleep disturbances and dementia risk: A multicenter study. Alzheimer’s Dement 14:1235–1242 doi:10.1016/j.jalz.2018.05.012 30030112 10.1016/j.jalz.2018.05.012

[69] Kivipelto M, Solomon A, Ahtiluoto S, et al (2013) The Finnish Geriatric Intervention Study to Prevent Cognitive Impairment and Disability (FINGER): Study design and progress. Alzheimer’s Dement 9:657–665 doi:10.1016/j.jalz.2012.09.012 23332672 10.1016/j.jalz.2012.09.012

[70] Zwan MD, Deckers K, Claassen JAHR, et al (2021) Study design of FINGER-NL: A multidomain lifestyle intervention in Dutch older adults to prevent cognitive decline. Alzheimer’s Dement. 10.1002/alz.055136 doi:10.1002/alz.055136

