# Supplementary material for: Daytime Sleepiness, Apnea, Neuroimaging Correlates and Cortisol Dysregulation in a Memory Clinic Cohort
Source: J Prev Alzheimers Dis. 2024 Jul 18;11(6):1798–808. doi: 10.14283/jpad.2024.145 (PMC11573860; doi:10.14283/jpad.2024.145)
Supplement: Supplementary file 1 — Supplementary material, approximately 27.2 KB. [file 42414_2024_356_MOESM1_ESM.docx]

# Supplementary material

Table S1 Associations between sleep disturbances and neuroimaging correlates.

Table S2 Associations between sleep disturbances and cortisol levels.

**Table S1** Associations between sleep disturbances and neuroimaging correlates.

|  | ***MTA (N=143)*** | | |  | ***GCA (N=140)*** | | |  | ***PA (N=73)*** | | |  | ***WMH (N=133)*** | | |
| --- | --- | --- | --- | --- | --- | --- | --- | --- | --- | --- | --- | --- | --- | --- | --- |
|  | *OR* | *95% CI* | *p* |  | *OR* | *95% CI* | *p* |  | *OR* | *95% CI* | *p* |  | *OR* | *95% CI* | *p* |
| **Sleep disturbances** |  |  |  |  |  |  |  |  |  |  |  |  |  |  |  |
| Total insomnia | *1.00* | *0.90 – 1.10* | *0.927* |  | *1.02* | *0.93 – 1.13* | *0.632* |  | *1.02* | *0.86 – 1.21* | *0.784* |  | *1.01* | *0.91 – 1.12* | *0.882* |
| Sleep quality | *0.96* | *0.88 – 1.05* | *0.428* |  | *0.97* | *0.88 – 1.06* | *0.506* |  | *0.94* | *0.80 – 1.10* | *0.444* |  | *1.05* | *0.96 – 1.16* | *0.300* |
| Sleep apnea | *0.89* | *0.76 – 1.03* | *0.125* |  | *0.92* | *0.77 – 1.09* | *0.374* |  | ***1.27*** | ***1.02 – 1.60*** | ***0.033*** |  | *0.95* | *0.79 – 1.14* | *0.570* |
| Daytime sleepiness (during work time) | *0.97* | *0.84 – 1.11* | *0.643* |  | *0.99* | *0.86 – 1.15* | *0.909* |  | *1.04* | *0.77 – 1.39* | *0.808* |  | *1.13* | *0.96 – 1.31* | *0.130* |
| Global sleep score | *0.98* | *0.95 – 1.00* | *0.107* |  | *1.00* | *0.98 – 1.03* | *0.782* |  | *1.02* | *0.97 – 1.06* | *0.385* |  | *1.00* | *0.97 – 1.03* | *0.931* |
| Initial insomnia | *0.95* | *0.76 – 1.18* | *0.643* |  | *1.03* | *0.82 – 1.30* | *0.782* |  | *1.09* | *0.74 – 1.58* | *0.670* |  | *1.01* | *0.80 – 1.28* | *0.919* |
| Middle insomnia | *0.97* | *0.78 – 1.21* | *0.766* |  | *1.02* | *0.81 – 1.28* | *0.879* |  | *0.96* | *0.63 – 1.45* | *0.855* |  | *0.91* | *0.72 – 1.14* | *0.410* |
| Terminal insomnia | *1.07* | *0.85 - 1.35* | *0.567* |  | *1.04* | *0.82 – 1.32* | *0.729* |  | *1.06* | *0.71 – 1.58* | *0.769* |  | *1.05* | *0.81 – 1.35* | *0.726* |
| Insufficient sleep | *0.97* | *0.77 – 1.22* | *0.798* |  | *1.16* | *0.91 – 1.49* | *0.224* |  | *1.06* | *0.71 – 1.58* | *0.757* |  | *1.29* | *0.99 – 1.68* | *0.058* |
| Difficulty waking up | *0.89* | *0.71 – 1.11* | *0.299* |  | *0.82* | *0.65 – 1.04* | *0.102* |  | *0.70* | *0.43 – 1.05* | *0.110* |  | *0.98* | *0.77 – 1.23* | *0.847* |
| Nightmares | *1.02* | *0.79 – 1.33* | *0.860* |  | *0.88* | *0.66 - 1.15* | *0.350* |  | *1.03* | *0.61 – 1.66* | *0.899* |  | *0.93* | *0.67 – 1.27* | *0.644* |
| Restless leg | *1.05* | *0.82 – 1.34* | *0.701* |  | *0.85* | *0.66 – 1.10* | *0.226* |  | *0.82* | *0.48 – 1.32* | *0.434* |  | *1.16* | *0.89 – 1.50* | *0.275* |
| Snoring | *0.97* | *0.79 – 1.20* | *0.810* |  | *0.95* | *0.75 – 1.18* | *0.635* |  | ***1.39*** | ***1.01 – 1.95*** | ***0.044*** |  | *1.08* | *0.84 – 1.39* | *0.528* |
| General sleep | *0.82* | *0.61 – 1.11* | *0.191* |  | *1.15* | *0.85 – 1.58* | *0.362* |  | *1.32* | *0.78 – 2.26* | *0.308* |  | *1.01* | *0.74 – 1.39* | *0.941* |

Ordered logistic regression models controlled for age, sex, education, and alcohol consumption. OR = odds ratio, MTA = medial temporal atrophy (Schelten’s scale), GCA = Global Cortical Atrophy (Pasquier scale), PA = Posterior atrophy (Koedam scale), WMH = White-matter Hyperintensities (Fazekas scale). All sleep measures are defined in Table 1 of the article. Higher sleep measures indicate worse sleep.

**Table S2** Associations between sleep disturbances and cortisol levels.

|  | ***Awakening cortisol*** | | |  | ***Bedtime cortisol*** | | |  | ***CAR*** | | |  | ***Total cortisol*** | | |  | ***Cortisol AM/PM ratio*** | | |
| --- | --- | --- | --- | --- | --- | --- | --- | --- | --- | --- | --- | --- | --- | --- | --- | --- | --- | --- | --- |
|  | $\beta$ | *95% CI* | *p* |  | $\beta$ | *95% CI* | *p* |  | $\beta$ | *95% CI* | *p* |  | $\beta$ | *95% CI* | *p* |  | $\beta$ | *95% CI* | *p* |
| **Sleep disturbances** |  |  |  |  |  |  |  |  |  |  |  |  |  |  |  |  |  |  |  |
| Total insomnia | -0.02 | -0.05 – 0.01 | 0.284 |  | 0.03 | -0.02 – 0.09 | 0.256 |  | -0.02 | -0.09 – 0.05 | 0.550 |  | -0.01 | -0.05 – 0.03 | 0.578 |  | -0.04 | -0.08 – 0.00 | 0.082 |
| Sleep quality | *-0.02* | *-0.05 – 0.01* | *0.131* |  | 0.02 | -0.03 – 0.07 | 0.515 |  | *0.01* | *-0.06 – 0.07* | *0.812* |  | *-0.01* | *-0.04 – 0.03* | *0.673* |  | *-0.03* | *-0.07 – 0.01* | *0.129* |
| Sleep apnea | ***-0.06*** | ***-0.11 - -0.01*** | ***0****.****012*** |  | -0.01 | -0.1 – 0.08 | 0.816 |  | *0.00* | *-0.11 – 0.11* | *0.988* |  | *-0.01* | *-0.07 – 0.05* | *0.715* |  | *-0.04* | *-0.10 – 0.02* | *0.214* |
| Daytime sleepiness  (during work) | *-0.03* | *-0.08 – 0.01* | *0.151* |  | 0.02 | -0.06 – 0.10 | 0.675 |  | *-0.06* | *-0.16 – 0.04* | *0.274* |  | *-0.03* | *-0.08 – 0.03* | *0.347* |  | *-0.04* | *-0.10 – 0.02* | *0.181* |
| Global sleep score | *0.00* | *-0.01 – 0.01* | *0.421* |  | 0.01 | -0.01 – 0.02 | 0.525 |  | *0.00* | *-0.02 – 0.02* | *0.800* |  | *0.00* | *-0.01 – 0.01* | *0.832* |  | *-0.01* | *-0.02 – 0.00* | *0.192* |
| Initial insomnia | *-0.04* | *-0.11 – 0.04* | *0.321* |  | 0.01 | -0.11 – 0.14 | 0.839 |  | *-0.04* | *-0.20 – 0.12* | *0.582* |  | -0.06 | -0.14 – 0.03 | 0.197 |  | -0.04 | -0.13 – 0.05 | 0.427 |
| Middle insomnia | *-0.07* | *-0.14 – 0.01* | *0.068* |  | 0.03 | -0.10 – 0.17 | 0.605 |  | *0.00* | *-0.17 – 0.16* | *0.984* |  | -0.02 | -0.11 – 0.07 | 0.616 |  | -0.07 | -0.16 – 0.03 | 0.165 |
| Terminal insomnia | *-0.02* | *-0.10 – 0.05* | *0.528* |  | 0.09 | -0.05 – 0.22 | 0.213 |  | *-0.09* | *-0.26 – 0.09* | *0.333* |  | -0.01 | -0.10 – 0.09 | 0.883 |  | -0.09 | -0.18 – 0.01 | 0.089 |
| Insufficient sleep | *-0.01* | *- 0.09 – 0.07* | *0.872* |  | 0.08 | -0.06 – 0.22 | 0.275 |  | *-0.02* | *-0.20 – 0.15* | *0.801* |  | *0.03* | *-0.07 – 0.12* | *0.568* |  | *-0.06* | *-0.16 – 0.04* | *0.242* |
| Difficulty waking up | *-0.03* | *-0.11 – 0.04* | *0.377* |  | 0.04 | -0.09 – 0.17 | 0.586 |  | *-0.06* | *-0.22 – 0.11* | *0.504* |  | *-0.01* | *-0.10 – 0.08* | *0.909* |  | *-0.05* | *-0.15 – 0.04* | *0.261* |
| Nightmares | *-0.05* | *-0.14 – 0.03* | *0.227* |  | 0.08 | -0.08 – 0.23 | 0.341 |  | *-0.14* | *-0.33 – 0.06* | *0.175* |  | *-0.03* | *-0.14 – 0.07* | *0.523* |  | *-0.09* | *-0.20 – 0.02* | *0.104* |
| Restless leg | *-0.07* | *-0.15 – 0.01* | *0.104* |  | -0.02 | -0.17 – 0.12 | 0.769 |  | *-0.04* | *-0.22 – 0.14* | *0.688* |  | *-0.07* | *-0.17 – 0.02* | *0.138* |  | *-0.04* | *-0.14 - 0.07* | *0.485* |
| Snoring | *-0.05* | *-0.12 – 0.02* | *0.178* |  | -0.05 | -0.18 - 0.09 | 0.487 |  | *-0.05* | *-0.21 – 0.12* | *0.564* |  | *-0.06* | *-0.15 – 0.02* | *0.150* |  | *0.00* | *-0.10 – 0.09* | *0.946* |
| General sleep | *-0.08* | *-0.18 – 0.02* | *0.097* |  | -0.05 | -0.22 – 0.13 | 0.601 |  | *-0.00* | *-0.22 – 0.22* | *0.987* |  | *-0.07* | *-0.19 – 0.05* | *0.223* |  | *-0.03* | *-0.15 – 0.10* | *0.669* |

Linear regression models controlled for age, sex, education, and alcohol consumption. CAR = Cortisol awakening response. All sleep measures are defined in Table 1 of the article. Higher sleep measures indicate worse sleep.
